# Supplementary material for: Enhanced subglacial discharge amplifies Petermann Ice Shelf melting when ocean thermal forcing saturates
Source: Nat Commun. 2025 May 6;16:4213. doi: 10.1038/s41467-025-59469-9 (PMC12056069; doi:10.1038/s41467-025-59469-9)
Supplement: Supplementary file 1 — Supplementary Information [file 41467_2025_59469_MOESM1_ESM.pdf]

Supplementary information for "Enhanced subglacial discharge amplifies Petermann Ice Shelf melting when ocean thermal forcing saturates"

Abhay Prakash[1,2,*], Qin Zhou[3], Tore Hattermann[4,5], and Nina Kirchner[1,2]

[1] *Department of Physical Geography, Stockholm University, Stockholm 10691, Sweden*
[2] *Bolin Centre for Climate Research, Stockholm University, Stockholm 10691, Sweden*
[3] *Akvaplan-niva, Tromsø 9296, Norway*
[4] *Norwegian Polar Institute, Tromsø 9296, Norway*
[5] *Complex Systems Group, Department of Mathematics and Statistics, UiT - The Arctic University of Norway, Tromsø 9019, Norway*
[*] *Corresponding author, abhay.prakash@natgeo.su.se*

A Model tuning

Table A1. Thermodynamic and ocean mixing parameters used in this study

Parameter	Value	Description
ρ_{fw}	1000 kg m^{-3}	Freshwater density
L	3.34×10^5 J kg^{-1}	Latent heat of fusion of ice
ρ_{sw}	1028 kg m^{-3}	Seawater density
c_w	3974 J °C^{-1} kg^{-1}	Specific heat capacity of seawater
Γ_T	1.2×10^{-2}	Non-dimensional heat-transfer coefficient
C_D	2.5×10^{-3}	Drag coefficient
u_{res}	1.0×10^{-2} m s^{-1}	Residual velocity
λ_1	-5.73 $\times 10^{-2}$ °C PSU^{-1}	Liquidus slope
λ_2	8.32 $\times 10^{-2}$ °C	Liquidus intercept
λ_3	-7.53 $\times 10^{-8}$ °C Pa^{-1}	Liquidus pressure coefficient
Γ_S	$\Gamma_T/35$	Non-dimensional salt-transfer coefficient
Z_o	1.0×10^{-3} [m]	Roughness length scale
Ro_{min}	2.5×10^{-3} [m]	Roughness minimum
K_m	1.0×10^{-5} [m^2 s^{-1}]	Vertical eddy viscosity
P_v	1.0	Vertical Prandtl Number
P_h	1.0×10^{-1}	Horizontal Prandtl Number
C_h	1.0×10^{-1}	Scaling constant

B Longitudinal ice shelf draft profile

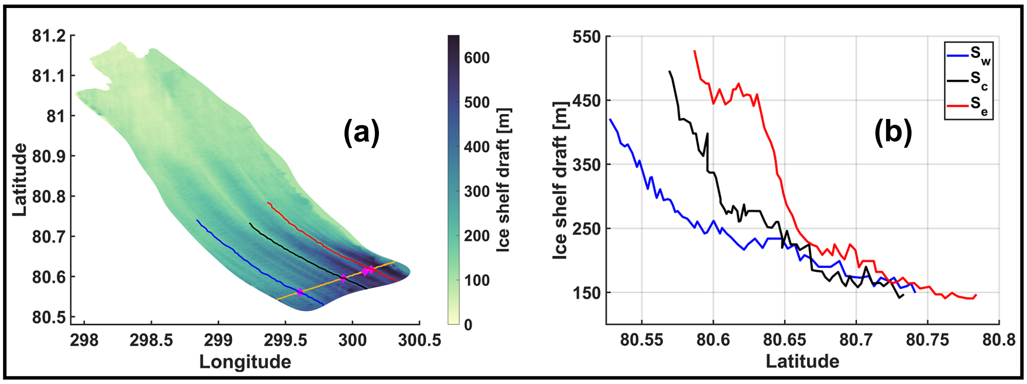

Figure B1. Map of the Petermann Ice Shelf draft (a) with the across-fjord transect ca. 5 km from the grounding line shown in Fig. 1(b) overlaid. Apex locations of four prominent longitudinal ice shelf basal channels at this transect (highlighted in Fig. 5) are represented using magenta diamonds. (b) Along-fjord profile of the Petermann Ice Shelf draft over the western (S_w; blue), central (S_c; black) and eastern (S_e; red) channel sections (Fig. 6(a)) as indicated in panel a.

C Indicators of a regime shift in heat flux efficiency beneath the Petermann Ice Shelf

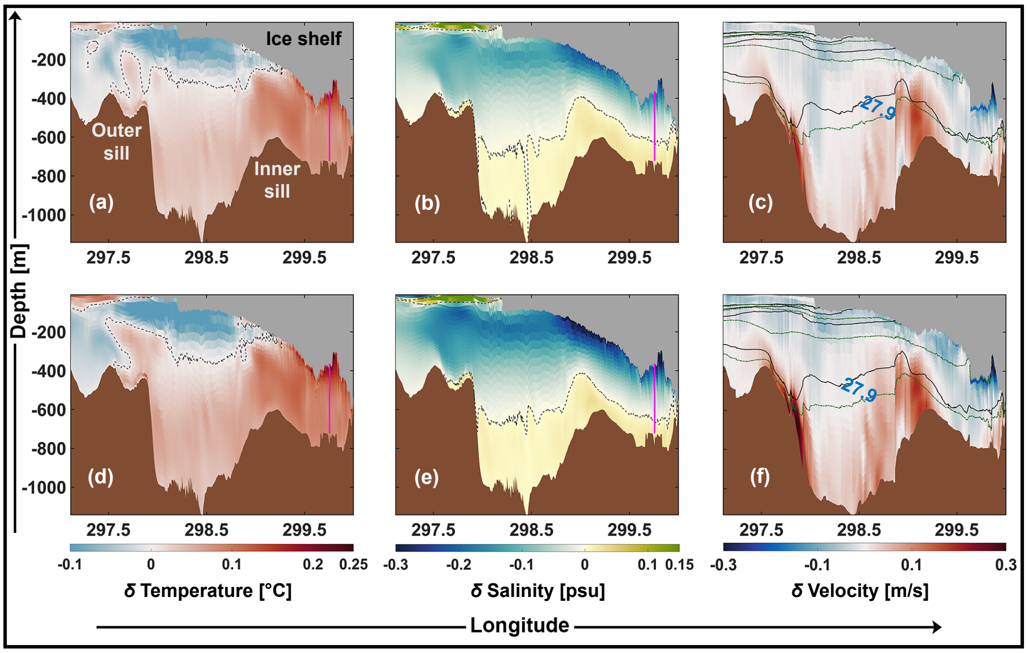

Figure C1. Along-fjord summer mean temperature (left column), salinity (middle column), and flow (right column) anomalies (δ) for the discharge (Q_{sg}) experiments Q_{sg}-median relative to control (first row) and Q_{sg}-RCP 8.5 relative to control (second row) for the section shown in Fig. 1(b). Black stippled lines in panels a, b, d and e indicate the zero temperature and salinity difference contours. Solid black lines in panels c and f depict the mean isopycnals for the control experiment, whereas the stippled green lines correspond to the Q_{sg}-median (c) and Q_{sg}-RCP 8.5 (f) experiments. Isopycnals are plotted at equal intervals of 0.2 kg/m^3. The 27.9 kg/m^3 isopycnal corresponds to the dense Atlantic Water that contacts the grounding line. In each panel, the grounding line (at ca. 600 m depth) is on the right margin and open ocean is to the left. Vertical magenta line in panels a, b, d, and e show the transect location closest to the model node ca. 10 km from the grounding line at which the vertical profile of summer mean temperature and salinity are shown for the control, Q_{sg}-present, Q_{sg}-median, and Q_{sg}-RCP 8.5 experiments in Fig. 3(g,h) (Table 1).

D Congruent patterns of increased freshening and melting

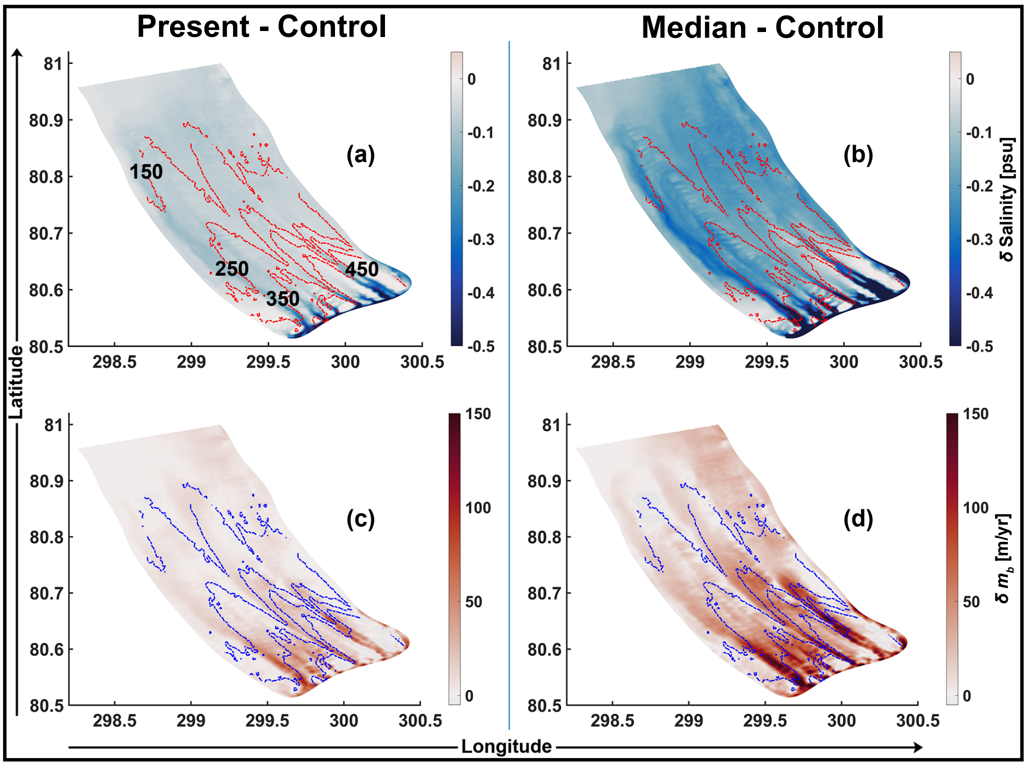

Figure D1. Anomaly (δ) maps of the summer mean surface layer salinity (a,b) and basal melt rate (m_b) (c,d) for the discharge (Q_{sg}) experiments Q_{sg}-present relative to control (column 1) and Q_{sg}-median relative to control (column 2). Contours of the ice shelf draft are plotted at 100 m intervals (450 m – 150 m; panel(a)) and overlaid as dotted red and blue lines over the surface layer salinity and melt rate maps, respectively.

E Modified sub-ice shelf water column thickness

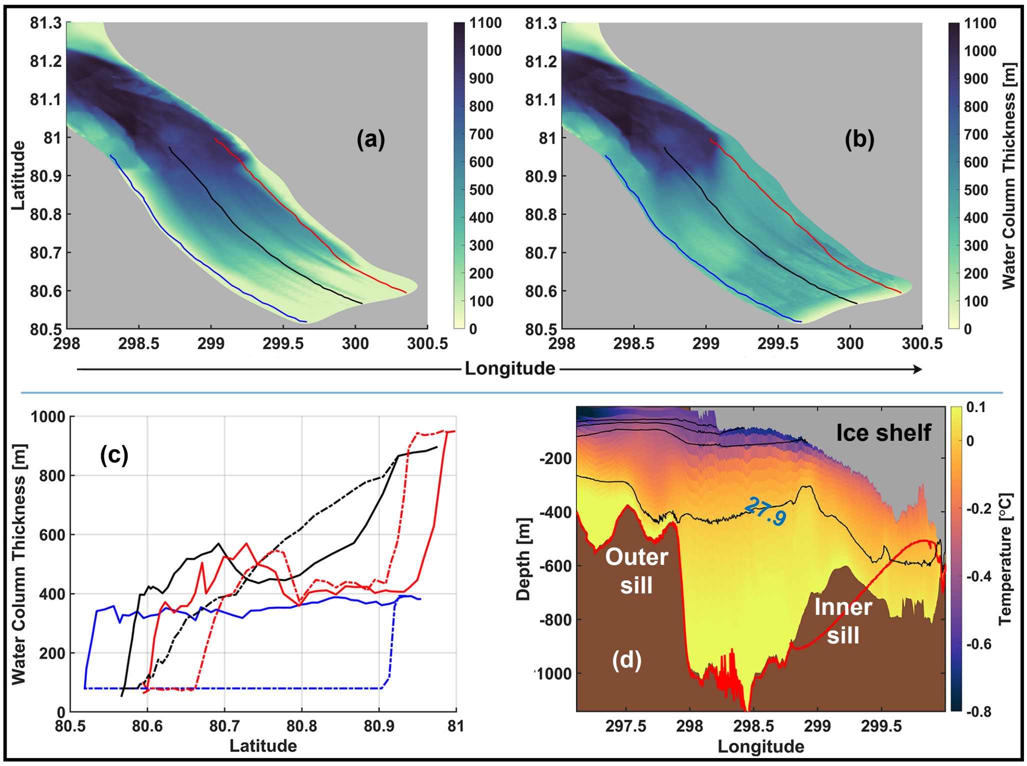

Figure E1. BedMachine v3 water column thickness (a), and modified BedMachine v3 water column thickness (b) used in this study. (c) Along-fjord profile of the water column thickness for the western (blue), central (black), and eastern (red) sections of the fjord as highlighted in panels (a) and (b). Stippled and solid lines correspond, respectively, to the BedMachine and modified BedMachine profiles. (d) Along-fjord summer mean temperature for the section shown in Fig. 1(b). Mean isopycnals (solid black lines) are overlaid at equal intervals of 0.2 kg/m^3. The 27.9 kg/m^3 isopycnal surface represents the dense Atlantic Water that contacts the grounding line. Solid red line represents the (unmodified) BedMachine v3 bathymetry, wherein, a synthetic sill blocks the 27.9 kg/m^3 isopycnal surface from contacting the grounding line.
